# HIV-Infected Patients Developing Tuberculosis Disease Show Early Changes in the Immune Response to Novel *Mycobacterium tuberculosis* Antigens

**DOI:** 10.3389/fimmu.2021.620622

**Published:** 2021-03-12

**Authors:** Noemi Rebecca Meier, Manuel Battegay, Tom H. M. Ottenhoff, Hansjakob Furrer, Johannes Nemeth, Nicole Ritz

**Affiliations:** ^1^University of Basel Children's Hospital, Mycobacterial Research Laboratory, Basel, Switzerland; ^2^University of Basel, Faculty of Medicine, Basel, Switzerland; ^3^Division of Infectious Diseases and Hospital Epidemiology, University Hospital Basel, University of Basel, Basel, Switzerland; ^4^Leiden University Medical Center, Department of Infectious Diseases, Leiden, Netherlands; ^5^Department of Infectious Diseases, Bern University Hospital, University of Bern, Bern, Switzerland; ^6^Division of Infectious Diseases, Zürich University Hospital, University of Zürich, Zurich, Switzerland; ^7^University of Basel Children's Hospital, Paediatric Infectious Diseases and Vaccinology Unit, Basel, Switzerland; ^8^Royal Children's Hospital Melbourne, Department of Paediatrics, University of Melbourne, Parkville, VIC, Australia

**Keywords:** T cell response, IGRA, RV, IP-10, TNF-alpha, Mycobacterium tuberculosis, TB, LTBI

## Abstract

**Background:** In individuals living with HIV infection the development of tuberculosis (TB) is associated with rapid progression from asymptomatic TB infection to active TB disease. Sputum-based diagnostic tests for TB have low sensitivity in minimal and subclinical TB precluding early diagnosis. The immune response to novel *Mycobacterium tuberculosis in-vivo* expressed and latency associated antigens may help to measure the early stages of infection and disease progression and thereby improve early diagnosis of active TB disease.

**Methods:** Serial prospectively sampled cryopreserved lymphocytes from patients of the Swiss HIV Cohort Study developing TB disease (“cases”) and matched patients with no TB disease (“controls”) were stimulated with 10 novel *Mycobacterium tuberculosis* antigens. Cytokine concentrations were measured in cases and controls at four time points prior to diagnosis of TB: T1-T4 with T4 being the closest time point to diagnosis.

**Results:** 50 samples from nine cases and nine controls were included. Median CD4 cell count at T4 was 289/ul for the TB-group and 456/ul for the control group. Viral loads were suppressed in both groups. At T4 Rv2431c-induced and Rv3614/15c-induced interferon gamma-induced protein (IP)-10 responses and Rv2031c-induced and Rv2346/Rv2347c-induced tumor necrosis factor (TNF)-α responses were significantly higher in cases compared to controls (*p* < 0.004). At T3 - being up to 2 years prior to TB diagnosis - Rv2031c-induced TNF-α was significantly higher in cases compared to controls (*p* < 0.004). Area under the receiver operating characteristics (AUROC) curves resulted in an AUC > 0.92 for all four antigen-cytokine pairs.

**Conclusion:** The *in vitro Mycobacterium tuberculosis*-specific immune response in HIV-infected individuals that progress toward developing TB disease is different from those in HIV-infected individuals that do not progress to developing TB. These differences precede the clinical diagnosis of active TB up to 2 years, paving the way for the development of immune based diagnostics to predict TB disease at an early stage.

## Background

Tuberculosis (TB) remains a major global health topic with an estimated quarter of the world's population being latently infected with *Mycobacterium tuberculosis* (1, 2). In 2019, an estimated 10 million people developed TB disease, killing 1.4 million people worldwide. Of those ~208,000 were co-infected with HIV (3). HIV-infected individuals are at particular risk of rapid progression from TB infection to subclinical and active TB disease (4, 5). Early detection, prediction of TB progression and treatment of TB infection and disease in these high-risk groups is therefore crucial to prevent disease progression and further transmission (6–9).

In the recent years, the binary perception of active versus latent TB has been replaced with the concept that TB is a spectrum of disease. After infection by *Mycobacterium tuberculosis* the pathogen may be cleared, persist, progress to disease in a slow or rapid fashion, or cycle through subclinical stages before developing into symptomatic TB disease (10, 11). Understanding of these dynamics is inherently difficult because clinical samples are typically collected only once disease has already developed. In this regard, the Swiss HIV Cohort Study, a systematic longitudinal study enrolling HIV-infected individuals in Switzerland, offers a unique opportunity to monitor the development of disease prior to the development of clinically apparent symptoms. We hypothesized that the analysis of the *Mycobacterium tuberculosis*-specific immune response during the time preceding clinical disease may inform on kinetics and pathophysiology involved in this process and may help to develop improved early diagnostic assays.

Currently, detection of TB relies almost entirely on sputum-based diagnostic assays which are likely to have lower sensitivity in minimal and subclinical TB disease (12). The most developed non-sputum-based assay used in HIV-infected individuals detects urine lipoarabinomannan but has a very limited sensitivity of 42% in patients with symptoms which further decreases in those with subclinical TB disease and higher CD4 counts (13). Blood-based diagnostic tests such as interferon-γ release assays (IGRA) using the RD1-*Mycobacterium tuberculosis* antigens early secretory antigen target (ESAT)-6, and culture filtrate protein (CFP)-10 have also a limited sensitivity of ~69% for detection of TB disease in HIV-infected individuals [pooled analysis from (14)]. In some studies the sensitivity of blood-based assays is even lower, as shown in a study within the framework of the Swiss HIV Cohort Study with 39% having a positive T-SPOT.TB within 6 months before culture-confirmed TB diagnosis (15).

Recent research suggest novel antigens may help to delineate the immune response preceding clinical disease as *Mycobacterium tuberculosis* changes its gene expression during infection and preceding clinical disease (16, 17). For example *Mycobacterium tuberculosis* antigens belonging to the group of latency associated antigens from the *Mycobacterium tuberculosis* dormancy of survival regulon (DosR) are of interest in the early and latent phases of infection (18). These genes are activated during the dormant non-replicative state and several studies show immune responses induced by these antigens to be more pronounced in latent TB infection compared to TB disease [reviewed in (16)]. Other antigens including heparin-binding haemagglutinin (HBHA) have also been used in blood tests for TB diagnosis (19, 20). In addition, the recently described *in-vivo* expressed *Mycobacterium tuberculosis* antigens, expressed in patients with pulmonary TB, have been found to elicit significant T cell responses (21). However, no study so far has been able to longitudinally investigate the immune response to these novel *Mycobacterium tuberculosis* antigens before the development of symptomatic TB disease.

The aim of this study was therefore to compare cytokine production after *in-vitro* stimulation with novel *Mycobacterium tuberculosis* antigens in HIV-infected patients up to 4 years prior to TB diagnosis. For this we used the prospectively collected and cryopreserved lymphocytes of the biobank of the Swiss HIV Cohort Study that allow longitudinal testing of the immune response prior to development of TB disease.

## Methods

### Study Design and Population

This case-control study was done within the framework of the Swiss HIV Cohort study which is a large cohort study in Switzerland which prospectively enrolls adult HIV-infected individuals. Demographic and clinical data including CD4 count, HIV viral load, antiretroviral treatment and screening and treatment of opportunistic infections are routinely collected, and blood samples for biobanking are taken at least annually (15).

**Figure 1:**
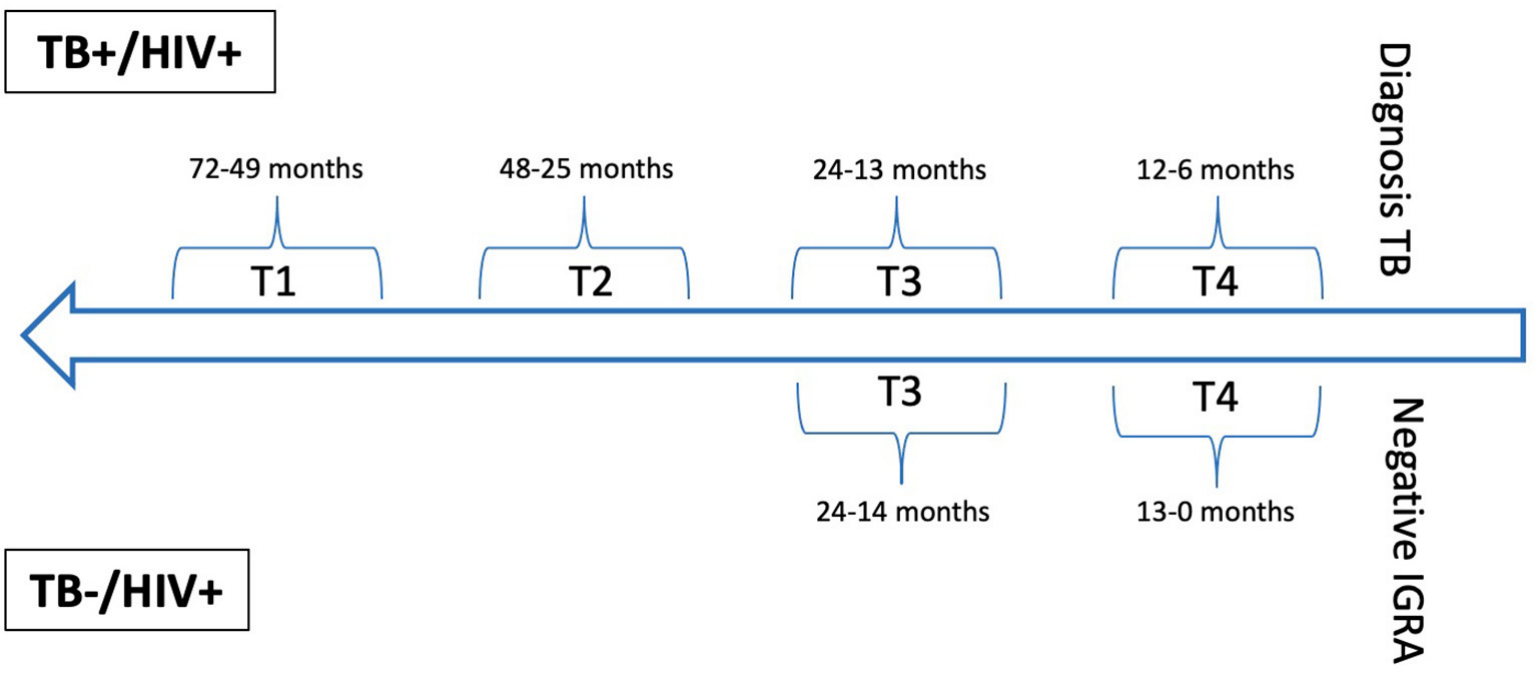
Study sampling timeline for TB and control group.

The database of the Swiss HIV Cohort study was searched for TB cases confirmed by culture or polymerase chain reaction and matched controls without a suspicion of TB and a negative IGRA. For cases annual or biennial blood samples (T1-T4), taken prior the diagnosis of TB disease were included. For controls two sequential annual blood samples (T3 and T4) were included, with T4 taken as close as possible to the negative IGRA (Figure 1, Supplementary Table 1). Matching was done for age, sex, body mass index, CD4 cell count, and HIV viral load.

### Viability of Frozen Lymphocytes, Sample Preparation, and Stimulation

For best possible conditions using frozen isolated peripheral blood mononuclear cells (PBMC) the following precautions were taken to ensure test performance: (i) sample processing in study centers according to harmonized protocol [as described in (15)] (ii) quality control of frozen samples in the framework of different studies [as described in (15)] and viability check of samples before the start of the assay (minimum of 70% recovery rate) (iii) sample selection from three study centers only (Basel, Berne, Zurich) to assure optimal cryopreservation and shipment and (iv) exclusion of samples that were stored before 2001.

Thawed lymphocytes (100,000 cells/condition) were stimulated overnight for 17 h at 37°C with the positive controls phytohemagglutinin (Merck chemicals LTD., Beeston, Nottingham, UK) at a concentration of 5 μg/ml and staphylococcus enterotoxin B (Sigma Aldrich GmbH, Schnelldorf, Germany) at a concentration of 10 μg/ml, novel *Mycobacterium tuberculosis* antigens (Rv0081, Rv1733c, Rv2031c, Rv0867c, Rv2389c, Rv3407, Rv2346/47c, Rv2431c, Rv3614/15c, and Rv3865) and a fusion protein of ESAT-6 and CFP-10 [all recombinant proteins expressed from *Escherichia coli* BL21 were produced by Kees L.M.C. Franken from the Ottenhoff lab at Leiden University Medical Center in the Netherlands (22)] at a concentration of 5 μg/ml and left unstimulated in the presence of CD28 and CD49d antibodies (Biolegend Inc., San Diego, Ca 92121, USA) at a concentration of 2 μg/ml each. The addition of costimulatory antibodies CD28 and CD49d has been done according to previously published protocols (23–25). After stimulation supernatants were stored at −20°C until further analysis. Experiments were done in a biosafety level 3^*^ facility.

### Cytokine Measurement

Granulocyte-macrophage colony-stimulating factor (GM-CSF), interferon gamma (IFN-γ), IFN-γ -inducible protein (IP)-10, interleukin (IL)-1RA, IL-6, and tumor necrosis factor (TNF)-α were measured using a human cytokine / chemokine magnetic bead panel (Milliplex MAP kit, Merck Millipore, Billerica, MA, USA), a Magpix Luminex instrument and Xponent software (version 4.2 Luminex Corp, Austin, Texas, USA) according to manufacturer's instructions. Standard curves using a 5-parameter logistic regression were applied to calculate concentrations of cytokines. Antigen- and mitogen-induced cytokine production was calculated by subtraction of non-stimulated background concentrations from sample concentrations. Measurements below the limit of quantification were set to 0.1 pg/ml, measurements above the limit of quantification were set to 10,000 pg/ml (calibration range: 3.2–10,000 pg/ml). A valid positive control was defined as an uncorrected cytokine concentration > 20 pg/ml (26). A valid negative control was defined as uncorrected cytokine concentration < 20 pg/ml. In cases where the nil concentration was higher than 20 pg/ml, the positive control had to be higher than the nil concentration.

### Statistical Analysis

Results from patients with invalid positive and negative control values were excluded from analysis for the specific time point and cytokine. If more than 25% of measurements from all patients for any cytokine were below the limit of quantification, the results from this cytokine were excluded from analysis. A Mann-Whitney *U*-test was used to compare differences in cytokine concentrations between the two groups at T3 and T4. Differences were considered significant if the *p*-value was < 0.004 (Bonferroni correction for multiple testing). Receiver operating characteristics (ROC) analyses were performed using area under the receiver operating characteristics (AUROC) where *p*-values were significant and optimal cut-off values determined. Differences in cytokine production between time points were calculated as absolute difference between T3 and T4 for controls and between T1, T2, and T3 to T4 for cases. All plots and statistical analyses were performed using R-studio software (version 1.1.463).

### Ethical Approval

The Swiss HIV Cohort Study was approved by the ethics committees of the different study centers and written consent was obtained from all participants.

## Results

### Study Population

A total of 50 samples from 18 individuals were included in the final analysis. Seven cases were male, median age was 45 (IQR 38–51) years, median CD4 cell count was 289 cells/μl, median RNA viral load was 16 copies/ml at T4 (Table 1). The median (range) time between TB diagnosis and T4 lymphocyte collection was 117 (29–312) days. In the controls seven were male, median age was 52 (IQR 41.5–56.5) years, median CD4 count was 456 cells/μl, median RNA viral load was 0 copies/ml at T4. The median (range) time between negative IGRA and T4 lymphocyte collection was 0 (0–420) days (Table 1). Age, sex, body mass index, CD4 count and RNA viral load were not significantly different between cases and controls. At T4 seven cases and eight controls were on antiretroviral treatment.

**Table 1:** Characteristics of study population.

**Variable**		**Tuberculosis group (T4)** ***N*** **=** **9**		**Control group (T4)** ***N*** **=** **9**	
		***n***	**%**	***n***	**%**
Median age, IQR (years)		45 (38–51)	-	52 (41.5–56.5)	-
Males		7	77.8	7	77.8
Median body mass index, IQR (kg/m^2^)		21.7 (20.5–23.2)	-	25.8 (24.1–27.7)	-
White ethnicity		6	66.7	9	100
TB disease	Pulmonary	4	44.4	-	-
	Extrapulmonary	4	44.4	-	-
	Pulmonary and extrapulmonary	1	11.1	-	-
Median CD4 cell count at TB diagnosis, IQR (cells/μl)		289 (152–422.5)	-	456 (258.5–601)	-
Median HIV-RNA at TB diagnosis, IQR (copies/ml)		16 (0–121,500)	-	0 (0–10,030)	-
Median time between cell sampling at T4 and TB diagnosis, range (days)		117 (29–312)	-	0 (0–420)	-
Median time between cell sampling at T3 and TB diagnosis, range (days)		440 (68–846)	-	392 (0–938)	-
Antiretroviral therapy at TB diagnosis		7	77.8	8	88.9
TST^*^	Negative	5	55.6	2	22.2
	>5–9 mm	0	0	0	0
	10–14 mm	0	0	1	11.1
	>15 mm	1	11.1	0	0
	Not done	3	33.3	6	66.7
IGRA	Negative	0	0	9	100
	Positive	1	11.1	0	0
	Indeterminate	0	0	0	0
	Not done	8	88.8	0	0
TB diagnosis^**^	Culture positive	3	33.3	-	-
	Sputum positive	4	44.4	-	-
	FNP PCR positive	1	11.1	-	-
	Lymphnode biopsy positive	1	11.1	-	-

* *TST, tuberculin skin test*.

** *One TB patient had a positive IGRA with clinical suspicion of but had no microbiological confirmation*.

### Antigen-Induced Cytokine Concentrations in Cases and Controls at T3 and T4

For the final analysis at T4 results from eight individuals were included for both study groups. At T3 results from seven individuals in the TB group and nine in the control group were included (Supplementary Table 2). GM-CSF, IFN-γ IL-6, IP-10, and TNF-α were detectable in most individuals in both study groups. Measurements for IL-1RA were commonly below the limit of quantification and therefore excluded from analysis (Supplementary Table 3).

#### Comparison of Results in TB Patients and Controls at Time Point Closest to Diagnosis (T4)

**Figure 2:**
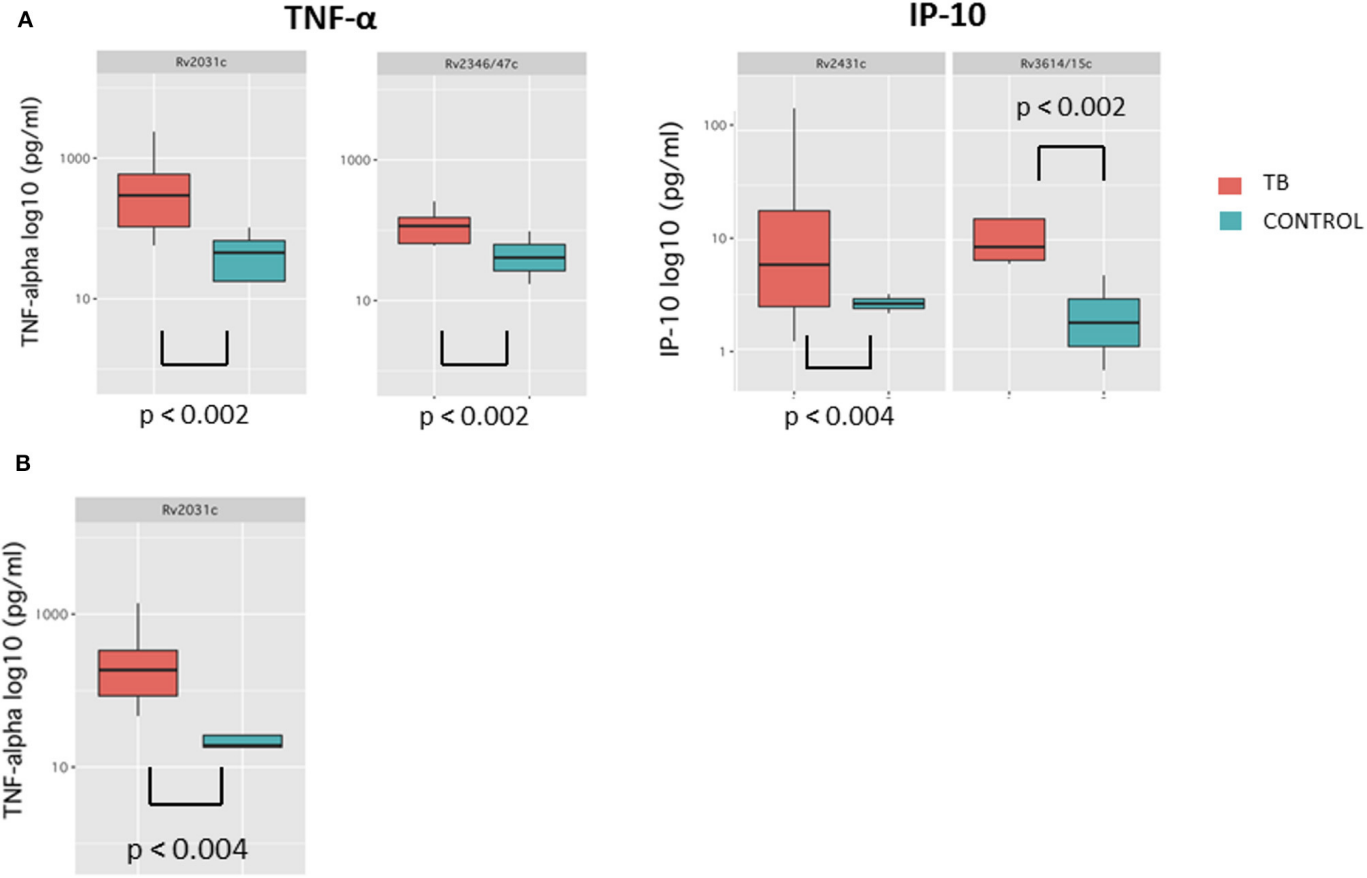
Box-and-whisker plots depicting median and interquartile range of cytokine concentrations results with significant differences at T4 **(A)** and T3 **(B)**.

Median cytokine concentrations in response to *Mycobacterium tuberculosis* antigens were generally higher in cases compared to controls at T4. These differences were most pronounced for IFN-γ, IP-10, and TNF-α and reached statistical significance for Rv2031c-induced TNF-α and Rv2346/47c-induced TNF-α (*p* < 0.002 for both) as well as for Rv2431c-induced IP-10 and Rv3614/15c-induced IP-10 (*p* < 0.004, *p* < 0.002, respectively) (Figure 2). Importantly, cytokine concentrations for the ESAT-6/CFP-10-induced IFN-γ were not significantly different in cases and controls at T4 (Supplementary Table 4).

#### Comparison of Results in TB Patients and Controls at Time Point 3 (T3)

Median cytokine concentrations in response to *Mycobacterium tuberculosis* antigens at T3 were generally higher in TB patients compared to controls (Figure 2). These trends were most pronounced for GM-CSF and TNF-α and reached significance for Rv2031c-induced TNF-α (*p* < 0.004). Median cytokine concentrations for the ESAT-6/CFP-10 -induced IFN-γ were also not significantly different in cases and controls at T3 (Supplementary Table 4).

#### Area Under the Receiver Operating Characteristics

**Figure 3:**
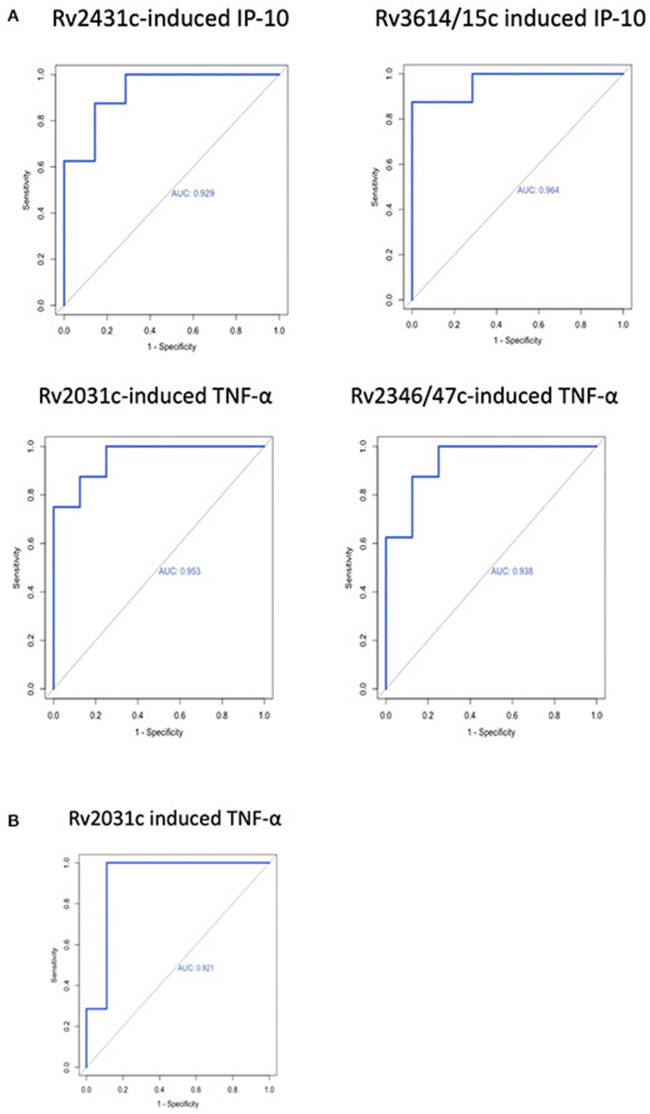
Receiver operating characteristic curves of antigen-cytokine pairs with significant difference between cases and controls at **(A)** T4 and **(B)** T3.

AUROC curves resulted in high AUC for the four antigen-cytokine pairs with significant differences between cases and controls: Rv2431c-induced IP-10 AUC 0.929 (95% CI = 0.800–1; T4); Rv3614/15c-induced IP-10 AUC 0.964 (95% CI = 0.881–1: T4); Rv2031c-induced TNF-α AUC 0.953 (95% CI = 0.862–1; T4) and 0.921 (95% CI = 0.76–1; T3); Rv2346/47c-induced TNF-α AUC 0.937 (95% CI = 0.824–1; T4). Cut-off concentrations were 2.2 pg/ml for Rv2431c-induced IP-10, 5.4 pg/ml for Rv3614/15c-induced IP-10, 72.9 pg/ml for Rv2031c-induced TNF-α, 25.3 pg/ml for Rv2346/47c-induced and 36.3 pg/ml for Rv2031c-induced TNF-α (Table 2 and Figure 3).

**Table 2:** Discriminatory potential of antigen-cytokine in cases and controls at T3 and T4.

**Antigen**	**Cytokine**	**TP**	**TB**	***n***	**Controls**	***n***	***p*-value**	**AUROC**	**Cut-off**	**Sensitivity (%)**	**Specificity (%)**
Rv2431c	IP-10	T4	6.0 (1.2–159.8)	8	−9.2 (−6.8- 3.2)	7	<0.004	0.929 (95% CI = 0.800–1)	2.2	0.87	1
Rv3614/15c	IP-10	T4	8.6 (0.6–238.8)	8	−0.7 (−9.3-4.7)	7	<0.002	0.964 (95% CI = 0.881–1)	5.4	0.87	1
Rv2031c	TNF-α	T4	304.1 (57.3-2381.0)	8	9.2 (-72.9-103.1)	8	<0.002	0.953 (95% CI= 0.862-1)	72.9	0.87	1
Rv2346/47c	TNF-α	T4	115.5 (6.6–1004.0)	8	−38.4 (-389.9-96.6)	8	<0.002	0.937 (95% CI = 0.824–1)	25.3	0.87	1
Rv2031c	TNF-α	T3	185.6 (46.5–1390.6)	7	2.6 (−814.1-447.7)	9	<0.004	0.921 (95% CI = 0.76–1)	36.3	1.0	0.89

*Median concentrations of cytokines (pg/ml) and ranges (in parenthesis) induced by stimulation of lymphocytes overnight and ability to discriminate between TB group and control group. AUROC, Area under the receiver operating characteristics*.

### Antigen-Induced Cytokine Concentrations Over Time

Differences in median cytokine concentrations between T3 and T4 in the controls were variable. Differences in GM-CSF, IFN-γ, and IP-10 concentrations were small for most stimulatory antigens except for ESAT-6/CFP-10 and Rv1733c. Larger differences in cytokine concentrations were observed for IL-6 and TNF-α. For IL-6 these differences were most pronounced following the with ESAT-6/CFP-10, Rv1733c, and Rv2389c (Supplementary Figure 1, Figure 5 show all data in normal scale for all cytokines).

**Figure 4:**
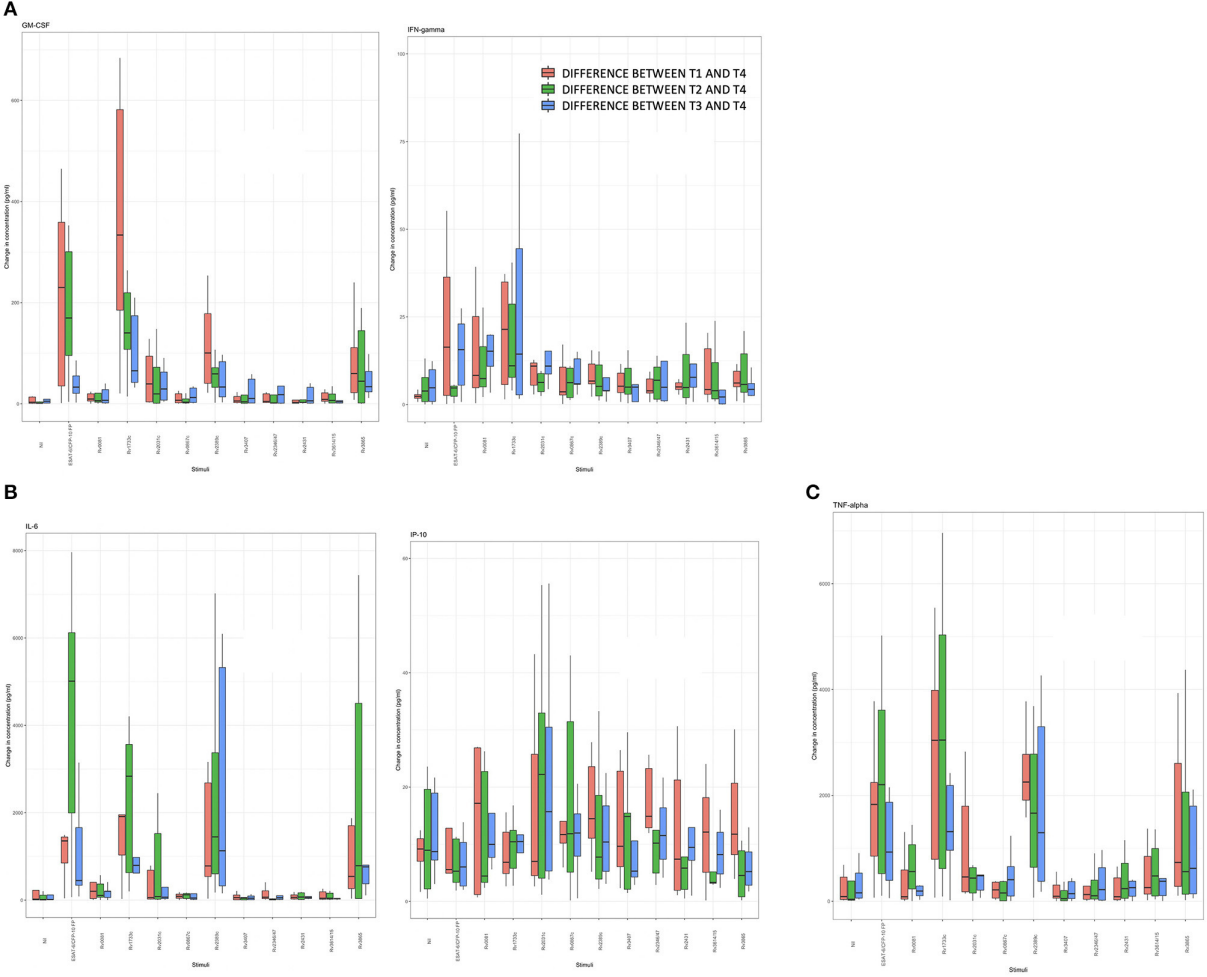
Difference of cytokine concentrations between T1, T2, T3, and T4 for the cases **(A–C)**.

**Figure 5:**
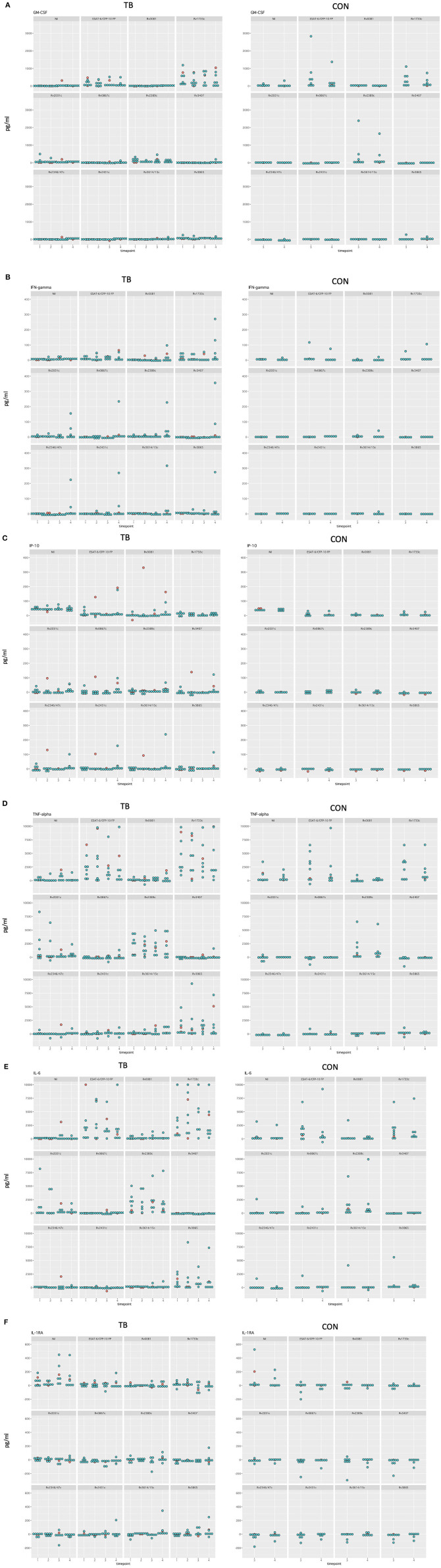
Dotplot of all cytokines in normal scale comparing TB and control group for all timepoints included **(A–F)**. Patients receiving antiretroviral treatment are color coded in red and patients not receiving antiretroviral treatment are color coded in blue.

Differences in median cytokine concentrations varied between T1, T2, T3, and T4 in the TB group and no clear pattern was detected (Figure 4). Generally, changes over time were more pronounced for IL-6 and TNF-α. Induced cytokine concentrations varied substantially for ESAT-6/CFP-10, Rv1733c, Rv2389c, and Rv3865 specifically for GM-CSF, IL-6, and TNF-α. Smaller differences in concentrations were seen in response to Rv0081, Rv0867c, Rv3407, Rv2346/47c, Rv2431c, and Rv3614/15c. Interestingly, differences in cytokine concentrations for the three *in-vivo* expressed antigens (Rv2346/47c, Rv2431c, and Rv3614/15c) over time were small among all cytokines (Figures 4, 5A–F showing all data in normal scale for all cytokines).

## Discussion

HIV-infected patients are at high risk of rapid progression from TB infection to TB disease. This study provides the first results in a unique setting of HIV-infected individuals with confirmed TB diagnosis in which their immune-response can be investigated prospectively during the stages developing TB disease prior to clinical diagnosis. The routine annual biobanking of lymphocytes allows analysis of potential early markers for minimal and subclinical TB disease. In the present study we show that several antigen-cytokine combinations clearly differentiate HIV-infected patients developing TB from those that do not develop TB up to 2 years prior to clinical diagnosis.

The three *in vivo*-expressed *Mycobacterium tuberculosis* antigens, Rv2346/47c, Rv2431c, and Rv3614/15c and the DosR antigen Rv2031c induced significantly higher TNF-α and IP-10 responses in TB patients shortly before clinical diagnosis compared to controls. Further to this our study found, that differences between patients developing TB and controls were already observed more than 12 months prior to diagnosis. Significantly different concentrations of TNF-α induced by Rv2031c were found between 1 and 2 years prior to clinical diagnosis of TB. Thus, this antigen-cytokine pair may serve as an early and antigen specifically induced correlate for TB infection and/or subclinical TB disease.

The potential of novel *Mycobacterium tuberculosis* antigens has mainly been studied for diagnostic purposes [summarized in (16)] or as novel vaccine candidates (21). So far, no study has assessed those antigens during development of TB disease. We included the latency associated antigens from the *Mycobacterium tuberculosis* DosR regulon which encodes for ~50 genes that are activated during the dormant non-replicative stage of TB (18). The DosR encoded antigens Rv0081, Rv1733c, and Rv2031c were used in this study based on evidence of previous studies that these are highly immunogenic (27–30).

Our results are in line with the few previous studies investigating this antigen. A study in a high TB-endemic setting with non-HIV-infected individuals described increased TNF-α concentrations induced by Rv2031c in patients with TB infection compared to controls (30). Important for this antigen is the role of measuring cytokines other than IFN-γ, as two other studies measuring IFN-γ only did not find significant differences in patients with TB disease, TB infection and controls (28, 31). A further study including a subgroup of four HIV-infected individuals also did not find noteworthy responses of IFN-γ induced by Rv2031c and two cytokines that were not measured in our study (IL-2, IL-17) (32).

The *in vivo*-expressed *Mycobacterium tuberculosis* antigens included in our study have not been studied in detail in humans. Research suggests that they are associated with virulence (Rv2346/47c, Rv2431c, Rv3614/15c, and Rv3865) (33), in particular Rv2346/47c, Rv3614/15c, and Rv3865 are all associated with the ESAT-6 secretion system. Since their absence in the Bacille Calmette-Guérin (BCG) vaccine strain, some of these antigens are of special interest in the diagnosis of previously immunized individuals (34). Data from our own study in children also showed the discriminatory potential of Rv2346/47c-induced IP-10 response in a population of HIV-negative children for TB infection and disease compared to exposed non-infected children (25). Further to this animal data showed the added value of Rv2346c when pooled with ESAT-6, CFP-10 and Rv3615c in a skin test in TB infected cattle (35).

Our study highlights the importance of antigen-induced TNF-α and IP-10 in the immune response during TB infection and developing disease. TNF-α seems to be key in HIV-TB coinfection with concentrations detected in this setting (36). Similar findings were seen in a South-African study using Quantiferon supernatants of HIV-infected individuals with TB and controls which showed that IP-10 was significantly different in stimulated and unstimulated samples between the groups (37). Generally, TNF-α is key for granuloma formation and recruitment of immune cells to the site of infection (38). We found TNF-α concentration to be significantly elevated in the TB group compared to the control group for two antigens. Several studies also show the discriminatory potential of TNF-α in diagnosing different stages of TB (23, 24, 39). IP-10 has been investigated in several studies (40, 41) also including HIV-infected individuals (42–45). In the current study and in our study using the same assays in children (25), *ex vivo* IP-10 responses significantly increased diagnostic accuracy if compared to the current standard testing.

Importantly, the current standard immunodiagnostic test—being IFN-γ-induced by ESAT-6/CFP-10—was unable to differentiate cases from controls at any time point. This suggests that the use of novel antigen-cytokine pairs is clearly needed to improve sensitivity to detect TB infection and disease in HIV-infected individuals. Due to its exploratory character this study included a limited set of samples. Despite this, clear trends in cytokine production induced by novel *Mycobacterium tuberculosis* antigens between cases and controls could be observed. The study setting prevented us from using fresh blood for the stimulation assays. To account for cryopreservation precautions were taken to minimize impact on assay performance. Some cytokines showed a high range of values and considerable variation over time. In our study setting we are unable to determine if this is true variation due to disease progression or variability.

## Conclusions

The *ex vivo Mycobacterium tuberculosis*-specific immune response of HIV-infected individuals developing TB disease is different form HIV-infected individuals without signs of TB infection. In line with our hypothesis, antigen specific responses were different prior to the clinical development of TB. These differences precede the clinical diagnosis of active TB up to 2 years, paving the way for the development of immune based diagnostics to predict early TB disease.

## Data Availability Statement

The raw data supporting the conclusions of this article will be made available by the authors, without undue reservation.

## Ethics Statement

The studies involving human participants were reviewed and approved by Ethikkommission beider Basel, Kantonale Ethikkommission Bern (21/88); Comité départemental d'éthique des spécialités médicales et de médecine communautaire et de premier recours, Hôpitaux Universitaires de Genève (01–142); Commission cantonale d'éthique de la recherche sur l'être humain, Canton de Vaud (131/01); Comitato etico cantonale, Repubblica e Cantone Ticino (CE 813); Ethikkommission des Kantons St. Gallen (EKSG 12/003); Kantonale Ethikkommission Zürich (KEK-ZH-NR: EK-793). The patients/participants provided their written informed consent to participate in this study.

## Members of the Swiss HIV Cohort Study

Aebi-Popp K, Anagnostopoulos A, Battegay M, Bernasconi E, Böni J, Braun DL, Bucher HC, Calmy A, Cavassini M, Ciuffi A, Dollenmaier G, Egger M, Elzi L, Fehr J, Fellay J, Furrer H, Fux CA, Günthard HF (President of the SHCS), Haerry D (deputy of “Positive Council”), Hasse B, Hirsch HH, Hoffmann M, Hösli I, Huber M, Kahlert CR (Chairman of the Mother & Child Substudy), Kaiser L, Keiser O, Klimkait T, Kouyos RD, Kovari H, Ledergerber B, Martinetti G, Martinez de Tejada B, Marzolini C, Metzner KJ, Müller N, Nicca D, Paioni P, Pantaleo G, Perreau M, Rauch A (Chairman of the Scientific Board), Rudin C, Scherrer AU (Head of Data Center), Schmid P, Speck R, Stöckle M (Chairman of the Clinical and Laboratory Committee), Tarr P, Trkola A, Vernazza P, Wandeler G, Weber R, Yerly S.

## Author Contributions

NM, NR, and MB developed the research question and the study design. TO provided the antigens. NM performed the experiments. NM and NR performed the data analysis. MB, TO, HF, and JN critically revised the analysis and the draft manuscript written by NM and NR. All authors reviewed and approved the final manuscript.

## Conflict of Interest

The authors declare that the research was conducted in the absence of any commercial or financial relationships that could be construed as a potential conflict of interest.

## Abbreviations

AUROC: area under the receiver operating characteristics
BCG: Bacille Calmette-Guérin
CFP-10: culture filtrate protein 10
CI: confidence interval
DosR: dormancy of survival regulon
ESAT-6: early secretory antigen target 6
GM-CSF: granulocyte-macrophage colony-stimulating factor
IFN-γ: interferon-gamma
IGRA: interferon-γ release assay
IL: interleukin
IP-10: INF-γ-inducible protein 10
ROC: receiver operating characteristics
TB: tuberculosis
TNF-α: tumor necrosis factor α
TST: Tuberculin skin test.
