# Supplementary material for: HIV-Infected Patients Developing Tuberculosis Disease Show Early Changes in the Immune Response to Novel Mycobacterium tuberculosis Antigens
Source: Front Immunol. 2021 Mar 12;12:620622. doi: 10.3389/fimmu.2021.620622 (PMC7994263; doi:10.3389/fimmu.2021.620622)

**Online Data Supplement**

**HIV-infected patients developing tuberculosis disease show early changes in the immune response to novel *Mycobacterium tuberculosis* antigens**

*N. R. Meier, M. Battegay, T.H.M. Ottenhoff, HJ. Furrer, J. Nemeth, N. Ritz and Swiss HIC Cohort Study*

**Table S1:** Interval in days between timepoints and diagnosis of TB (cases) and negative test results (controls).

| TB cases | T1 | T2 | T3 | T4 |
| --- | --- | --- | --- | --- |
| TB1 | 1095 | 256 | 711 | 79 |
| TB2 | 1635 | 1279 | 68 | 312 |
| TB3 | 1127 | 777 | 440 | 117 |
| TB4 | 981 | 772 | 386 | 29 |
| TB5 | 1171 | 924 | 567 | 182 |
| TB6 | 1191 | 980 | 596 | 119 |
| TB7 | 1580 | 1217 | 846 | 145 |
| TB8 | 1113 | 691 | 299 | 98 |
| TB9 | 1297 | 953 | 406 | 99 |

| Controls | T3 | T4 |
| --- | --- | --- |
| CON1 | 175 | 0 |
| CON2 | 397 | 0 |
| CON3 | 394 | 0 |
| CON4 | 448 | 0 |
| CON5 | 308 | +182 |
| CON6 | 0 | +420 |
| CON7 | 366 | 0 |
| CON8 | 392 | 0 |
| CON9 | 938 | 0 |

**Table S2:** Number of included results listed for study groups, time points and cytokines.

|  | **TB group (n=9)** | | | | **Control group (n=9)** | |
| --- | --- | --- | --- | --- | --- | --- |
|  | **T1** | **T2** | **T3** | **T4** | **T3** | **T4** |
| GM-CSF | 9 | 9 | 7 | 8 | 9 | 8 |
| IFN-γ | 9 | 9 | 7 | 8 | 7 | 6 |
| IL-6 | 9 | 8 | 7 | 8 | 9 | 8 |
| IP-10 | 9 | 8 | 7 | 8 | 7 | 7 |
| TNF-α | 9 | 9 | 7 | 8 | 9 | 8 |
| IL-1RA * | 0 | 0 | 0 | 0 | 0 | 0 |
| Total | 45 | 43 | 35 | 40 | 41 | 37 |

* Measurements for IL-1RA were commonly below the limit of quantification and therefore excluded from analysis (**supplementary table S2**).

**Table S3:** Proportion of cytokine concentrations below the

limit of quantification (BLQ) overall and per study group for

all time points (T1-T4)

| ***Cytokines*** | ***Overall  (% BLQ)*** | ***TB***  ***(% BLQ)*** | ***CON  (% BLQ)*** |
| --- | --- | --- | --- |
| **GM-CSF** | 2.0 | 0.0 | 6.5 |
| **IFN-γ** | 7.0 | 0.9 | 20.9 |
| **IL-6** | 7.0 | 6.2 | 8.8 |
| **IP-10** | 0.0 | 0.0 | 0.0 |
| **TNF-α** | 0.1 | 0.1 | 0.0 |
| **IL-1ra** | 26.0 | 15.6 | 49.7 |
| IL = Interleukin, IP = IFN-γ-inducible protein, IFN = Interferon, TNF = Tumor necrosis factor , IL-1ra: IL-1 receptor antagonist, GM-CSF = Granulocyte-macrophage colony-stimulating factor | | | |

**Table S4**: p-values for Mann-Whitney U test between TB and control group at time points T4 and T3 (N=18)

|  | *Nil* | *ESAT-6/CFP-10 fusion protein* | *Rv0081* | *Rv1733c* | *Rv2031c* | *Rv0867c* | *Rv2389c* | *Rv3407* | *Rv2346/47c* | *Rv2431c* | *Rv3614/15c* | *Rv3865* |
| --- | --- | --- | --- | --- | --- | --- | --- | --- | --- | --- | --- | --- |
| **T4** | | | | | | | | | | | | |
| GM-CSF | 0.0650 | 0.4418 | 0.8785 | 0.7209 | 0.1889 | 0.3717 | 0.7984 | 0.0208 | 0.0273 | 0.0156 | 0.1049 | 0.6454 |
| IFN-γ | 0.2284 | 0.4908 | 0.1079 | 0.1812 | 0.0451 | 0.1079 | 0.1419 | 0.1812 | 0.1079 | 0.1812 | 0.345 | 0.4136 |
| IL-6 | 0.1267 | 0.2345 | 0.8335 | 0.1605 | 0.3823 | 0.2933 | 0.4418 | 0.7923 | 0.0585 | 0.5992 | 0.1719 | 0.3823 |
| IP-10 | 0.3969 | 0.0541 | 0.0939 | 0.6943 | 0.0093 | 0.3969 | 0.0205 | 0.0205 | 0.0205 | **0.0037** | **0.0012** | 0.0093 |
| TNF-α | 0.0650 | 0.5737 | 0.7984 | 0.1304 | **0.0011** | 0.0830 | 0.6454 | 0.0650 | **0.0019** | 0.2345 | 0.0650 | 0.1605 |
| **T3** | | | | | | | | | | | | |
| GM-CSF | 0.3968 | 0.6806 | 0.2523 | 0.536 | 0.0549 | 0.7506 | 0.9182 | 0.2105 | 0.0711 | 0.9182 | 0.1416 | 0.0115 |
| IFN-γ | 0.1649 | 0.3176 | 0.9015 | 1 | 0.6077 | 0.3176 | 0.3829 | 0.7104 | 0.8982 | 0.4817 | 0.3062 | 0.1792 |
| IL-6 | NA | 0.4698 | 0.351 | 0.1416 | 0.0549 | 0.4574 | 0.0907 | 0.9155 | 0.2991 | 1 | 0.7501 | 0.0311 |
| IP-10 | 0.1282 | 0.9015 | 1 | 0.4557 | 1 | 0.7104 | 0.3829 | 0.535 | 0.4557 | 1 | 0.535 | 0.0530 |
| TNF-α | 0.536 | 0.9182 | 0.1142 | 0.2523 | **0.0033** | 0.4079 | 0.2523 | 0.2105 | 0.1416 | 0.2523 | 0.0418 | 0.0311 |

**Figure S1:** Absolute difference of median cytokine concentrations between T3 and T4 for the control group


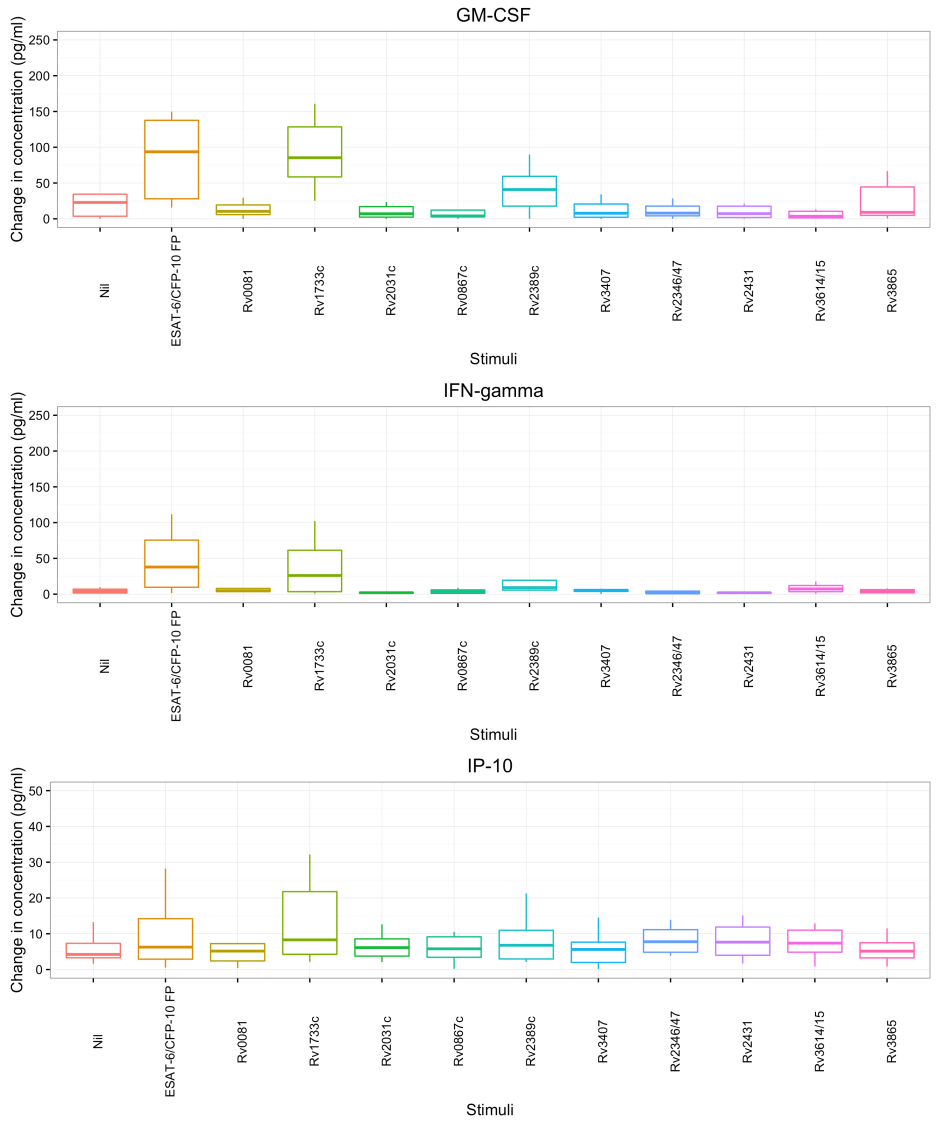

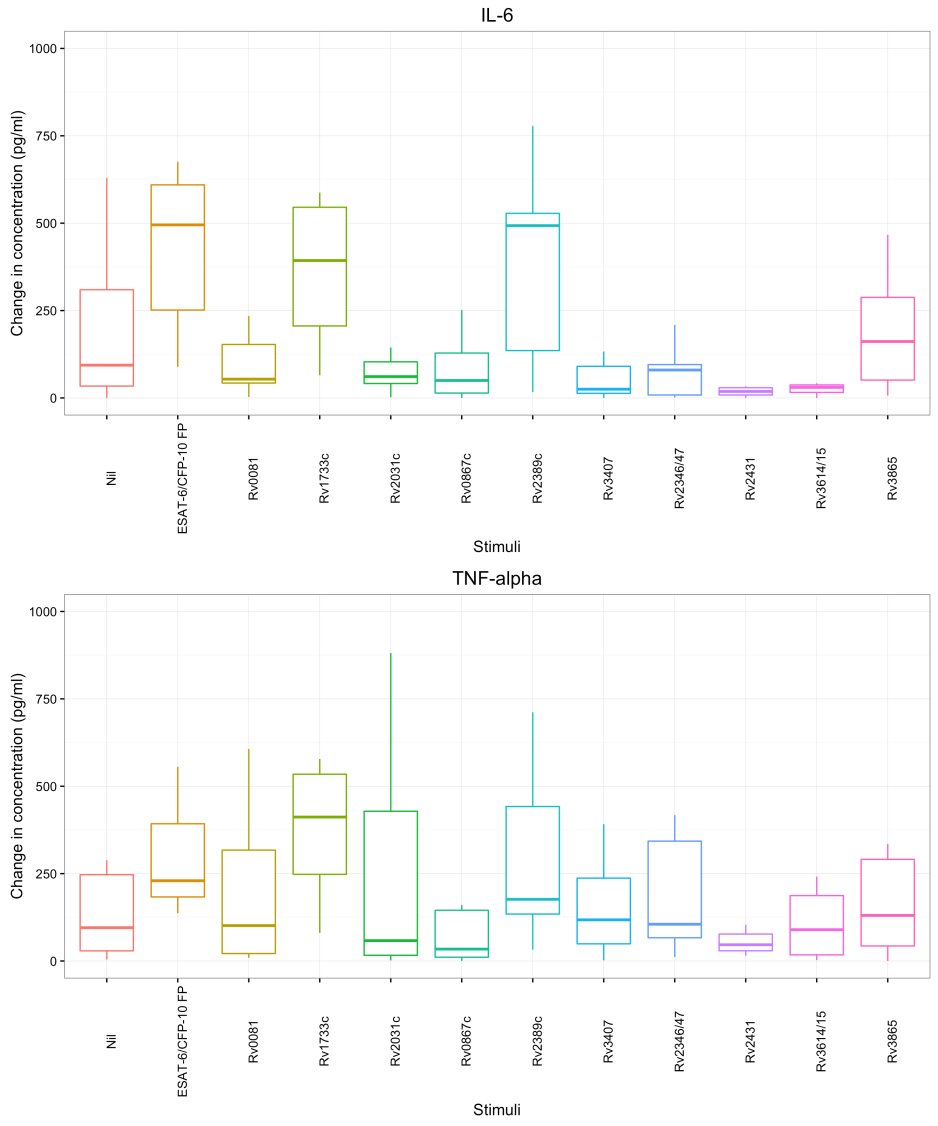

Supplement: Supplementary file 1 [file Data_Sheet_1.docx]
